# MRSA prevalence among patient transport staff in Hamburg

**DOI:** 10.3205/dgkh000309

**Published:** 2018-03-13

**Authors:** Anja Schablon, Olaf Kleinmüller, Albert Nienhaus, Claudia Peters

**Affiliations:** 1 Institute for Health Services Research in Dermatology and Nursing, University Medical Center Hamburg-Eppendorf, Hamburg, Germany; 2 Department of Occupational Medicine, Public Health and Hazardous Substances, Statutory Accident Insurance and Prevention in the Health and Welfare Services, Hamburg, Germany

**Keywords:** patient transport employees, MRSA, colonization, infection control

## Abstract

**Introduction:** Patient transport employees frequently come into contact with multidrug-resistant organisms (MDROs) and therefore are at a greater risk of infection than the general population. These pathogens pose a significant challenge for employees of patient transport services since they can spread over long distances through patient transfers. To date, little is known about the occupational risk of MRSA infection in patient transport settings.

**Methods:** A cross-sectional study was conducted to investigate the prevalence of MRSA in patient transport personnel, including taxi drivers, as well as the potential risk factors for MRSA colonization. For screening, nasal swabs were taken. When an individual was tested positive, a control swab was taken; if this confirmed a positive result, decolonization measures were offered. A molecular biological examination of the MRSA samples was performed.

**Results:** A total of 222 patient transport employees were screened and 7 employees tested positive, putting the MRSA prevalence at 3.2% (95% CI 1.4–6.5). Significant risk factors among patient transport staff (PTS) for testing positive were the use of antibiotics (OR 11.9; 95% CI 1.8–78.4) and hospital admission (OR 6.9; 95% CI 1.1–45.9). MRSA swabs were also performed on a total of 102 taxi drivers who provide patient transport services. The MRSA prevalence was 0.98 (95% CI <0.01–5.9). Significant group differences between PTS and taxi drivers, with respect to potential risk factors for MRSA colonization, were identified as inpatient treatment (p=0.09), chronic respiratory illnesses (p=0.01), and knowingly transporting patients/passengers with MRSA (p=0.03).

**Conclusion:** This study is the first to make data on the MRSA risk of patient transport employees in Hamburg available. The prevalence data are low in all areas and indicate a somewhat low risk of infection. A good infection control at the facilities is highly recommendable and the employees should acquire in-depth knowledge of infection prevention to improve compliance with personal protective measures.

## Zusammenfassung

**Einleitung:** Beschäftigte im Krankentransport kommen häufig in Kontakt mit Krankheitserregern und haben durch ihre berufliche Tätigkeit ein erhöhtes Infektionsrisiko im Vergleich zur Allgemeinbevölkerung. Multiresistente Erreger (MRE) stellen für das Personal von Krankentransportunternehmen eine besondere Herausforderung dar, da Krankheitserreger durch die Patiententransporte über weite Strecken verbreitet werden können. Bislang ist wenig über das berufsbedingte Infektionsrisiko bei Beschäftigten im Krankentransport bekannt.

**Methode:** In einer Querschnittstudie wurden die MRSA-Prävalenz und mögliche Risikofaktoren für eine Besiedlung bei Beschäftigten im Krankentransport (KTP) einschließlich Taxifahrern untersucht. Das Screening erfolgte durch Nasenabstriche. Bei einem positiven Testergebnis wurde ein Kontrollabstrich angeboten, bei erneut positivem Test eine Dekolonisationsmaßnahme. Bei positiven Proben erfolgte eine Genotypisierung.

**Ergebnisse:** Insgesamt wurden 222 Beschäftigte aus Krankentransportunternehmen untersucht; bei 7 fand sich ein positives Ergebnis. Das entspricht einer MRSA-Prävalenz von 3,3%. Signifikante Risikofaktoren für eine Besiedlung waren die Einnahmen von Antibiotika (OR 11,9, 95% KI 1,8–78,4) und ein vorheriger Krankenhausaufenthalt (OR 6,9, 95% KI 1,1–45,9). Bei 102 Taxifahrern, die Krankenfahrten durchführten, betrug die MRSA-Prävalenz 0,98% (n=1). Signifikante Unterschiede von Risikofaktoren für eine Besiedlung zwischen den beiden Gruppen fanden sich für Krankenbehandlung (p=0,09), chronische Atemwegserkrankungen (p=0,01) und wissentliche Transporte von Patienten/Fahrgästen mit MRSA (p=0,03).

**Schlussfolgerung:** Die MRSA-Prävalenz beim KTP ist niedrig und spricht eher für ein geringes Infektionsrisiko. Wichtig ist ein gutes Hygienemanagement, ein fundiertes Wissen der Beschäftigten über das Thema Infektionsschutz sowie Informationen und Kommunikation zum Infektionsstatus des Patienten, um geeignete Schutzmaßnahmen treffen zu können.

## Introduction

Patient transport employees frequently come into contact with pathogens and therefore are at a greater risk of infection than the general population due to their occupation. Multidrug-resistant organisms (MDROs) are increasingly becoming a public health problem. Methicillin-resistant *Staphylococcus aureus* (MRSA), which is prevalent around the world, is the best known MDRO. This pathogen also poses a significant challenge for the employees of patient transport services since it can spread over long distances through patient transport [1]. 

Analyses conducted by the Statutory Accident Insurance and Prevention in the Health and Welfare Services (BGW) show that the risk of MRSA infection is relevant to healthcare workers. Between 2010 and 2014, a total of 263 suspected cases of occupational illness caused by MRSA were reported. In the same period, 39 cases were confirmed. Only MRSA infections contracted during activity that is associated with an increased risk of infection are recognized as an occupational disease [2]. 

Patient transportation services play an important role in transferring patients and represent an important link between the various healthcare facilities. A distinction is made between qualified and non-qualified patient transport. For qualified patient transport, there are guidelines for good hygiene to prevent the spread of infection which are established by the laws pertaining to rescue services in the individual German states. Data on the prevalence of MRSA among paramedics in Germany are not known. The existing data were collected in the US. In Ohio, a prevalence rate of 4.6% was found among patient transport staff [3]. Studies in Germany deal with the microbial load of vehicles and medical devices; the employees themselves have not been tested until now [4], [5]. 

Despite the occupational risk posed by MRSA to patient transport employees, patient transport has not been systematically included in monitoring until now. Routinely collected data would be required in order to better assess the exposure risk of employees. 

This cross-sectional study was conducted to examine the prevalence of MRSA in patient transport staff (PTS) and the potential risk factors for MRSA colonization in Hamburg.

## Methods

In order to describe the contamination situation of MRSA among paramedics and taxi operators providing patient transport, a cross-sectional study on the point prevalence of MRSA colonization was carried out in Hamburg in 2016. A total of 18 facilities, companies, and organisations that provide qualified and non-qualified patient transport services, as well as seven companies that only provide non-qualified patient transport, were contacted. Initial contact was made via email and letter. The facilities that did not respond to the email/letter campaign were then contacted via telephone. The goal was to recruit as many facilities as possible for MRSA screenings of the employees in their local departments. In addition, flyers were distributed announcing dates at the University Medical Center Hamburg-Eppendorf (UKE) when patient transport staff could participate independent of the dates for their departments. The participating taxi drivers were recruited in cooperation with the German Social Accident Insurance Institution for Commercial Transport, Postal Logistics and Telecommunication (BG Verkehr). BG Verkehr employees directly contacted the respective taxi firms and control centers that they supervise. Here, the goal was also to conduct the screenings on-site at the companies where possible. Flyers were used to advertise the additional dates for the swab tests at the UKE.

Swabs from the nasal vestibules of the employees were taken for the purpose of this screening. Potential risk factors for MRSA colonization were identified using a questionnaire. Occupational risk factors, such as the nature and duration of their work and contact with MRSA patients, as well as other influences, such as taking antibiotics, their own hospital stays, and contact with animals, were explored alongside socio-demographic data. Being older than 18 years of age was set as an inclusion criterion. MRSA diagnosis was performed by testing for *S. aureus* and specific MRSA resistance to methicillin. In the case of positive samples, a molecular biological typing procedure was performed (*S. aureus* protein A gene (*spa*) typing). All analyses were performed in accordance with the available quality standards. If MRSA findings were positive, the employees were first given the option of a control swab. If the result from this control swab was still positive, decolonization measures were taken. Decolonization kits were provided to those affected. A further control swab was offered to check whether the decolonization efforts had been successful. The study was conducted in accordance with the requirements of data protection legislation. The Hamburg Ethics Commission gave its approval.

The univariate analyses were performed using Pearson’s chi-square tests or if cell frequency was low using Fisher’s exact test. Persons who tested positive for MRSA were compared against persons who tested negative. For the multivariate analysis, logistic regression was applied. The analyses were performed using IBM SPSS Statistics 23.

## Results

### Patient transport

A total of 222 emergency medical service workers were screened and 7 employees tested positive, resulting in an MRSA prevalence of 3.2% (95% CI 1.4–6.5) (Table 1 (Tab. 1)). Trained rescue workers made up 6.3% of the employees, assistant paramedics constituted 27.5% and paramedics comprised the largest share, with 46.8%. Additional occupations mentioned were emergency paramedics (6.8%), interns/trainees (5.9%), and other professions (6.8%). When stating their workplace, 68% said that they worked in an ambulance, followed by patient transport (30.2%), transport services for people with disabilities (3.6%), intensive care transport (2.3%), and ambulance (5.9%). The age of the youngest employee was 18 years and the oldest was 67. The median age was 32. The proportion of women was 21.4% (n=47). When asked about potential risk factors, 6.8% of the participants cited their own hospitalization within the last 12 months and 19.4% cited the use of antibiotics within the last 6 months. Other potential risk factors are listed in Table 1 (Tab. 1).

The opportunity to obtain a control swab was taken by 6 of the 7 subjects who tested positive. These control swabs yielded 3 positive results and 3 negative results. The 3 participants who tested positive after the control swab underwent decolonization treatment, which was not successful for one employee. This employee was referred to the responsible occupational physician.

**Figure 1:**
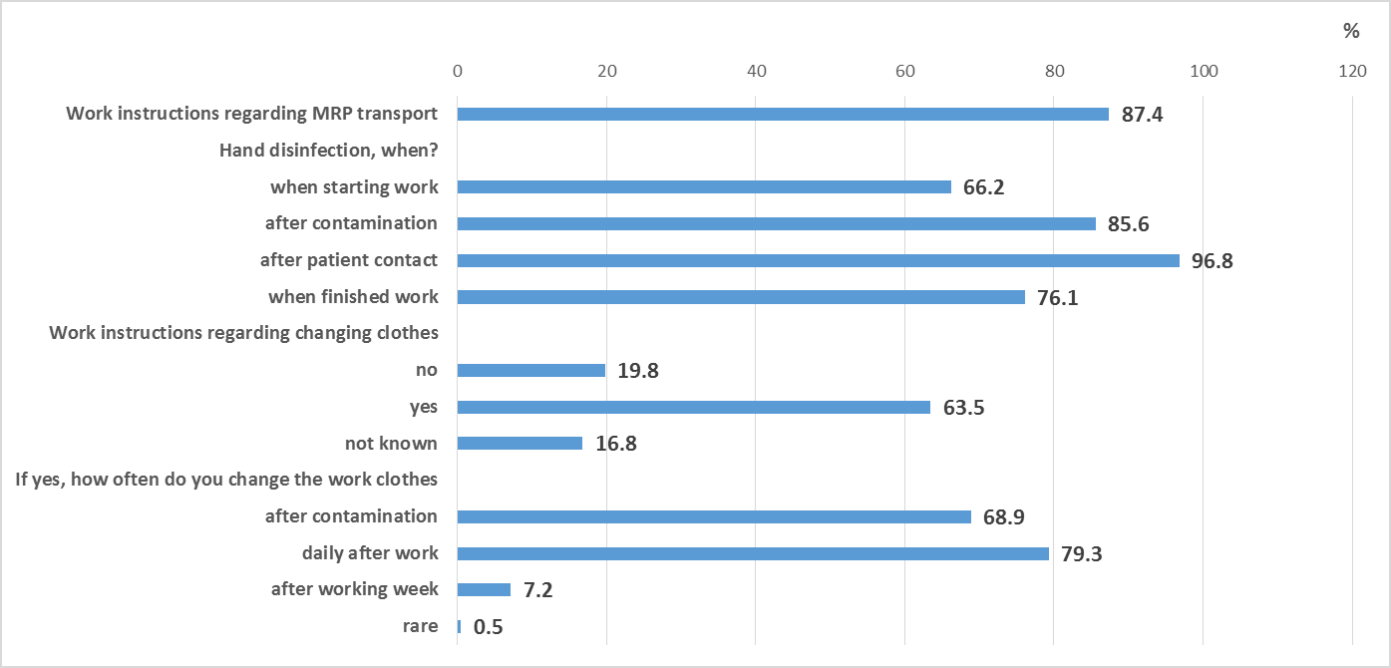
Questions about occupational safety in patient transport

87.4% of PTS personnel reported being familiar with work instructions on how to deal with MRSA/MDRO and 75.2% knowingly transported patients with MRSA in the past year (see Table 1 (Tab. 1)). In response to other questions regarding occupational safety, 97% of the employees said that they disinfect their hands after patient contact. 85% disinfected their hands after contamination. Work instructions regarding changing clothes were known to 63.5% of the employees (Figure 1 (Fig. 1)).

Significant risk factors among PTS personnel for testing positive were the use of antibiotics (OR 11.9; 95% CI 1.8–78.4) and hospital admission (OR 6.9; 95% CI 1.1–45.9) (Table 2 (Tab. 2)).

### Taxis

MRSA swabs were performed in a total of 102 taxi drivers who provide patient transport services. The MRSA prevalence was 0.98 (95% CI < 0.01–5.9). The median age of the taxi drivers was 53 years. The youngest participant was 23 years old and the oldest was 74. The proportion of women was 19.2%. 51% of the taxi drivers had been working in the profession for more than 10 years. 97% said that they had knowingly transported clients with MRSA, but only 6.9% (n=7) reported that there were instructions in their company for the transport of passengers with MRSA (Table 1 (Tab. 1)). 

When asked about potential risk factors for a positive MRSA swab, 12.7% (n=13) of the taxi drivers reported having been hospitalized for treatment in the last 12 months and 14.7% had been given antibiotics in the last 6 months. A total of 3 taxi drivers cared for relatives in their home or for relatives/retirement home residents as an additional occupation or worked at the volunteer fire department. To the question of whether they disinfect their hands before or after transporting passengers, 30.4% of the participants answered yes, 46.1% answered no, and 20.6% only did this if they knew the passenger had an illness (data not shown).

With respect to potential risk factors for MRSA colonization, significant group differences between emergency medical service workers and taxi drivers were identified as inpatient treatment (p=0.089), chronic respiratory illnesses (p=0.002) and knowingly transporting patients/ passengers with MRSA (p=0.028) (Table 1 (Tab. 1)).

### Genotyping

The genotyping of MRSA samples showed widespread MRSA strains, particularly in Germany and Europe. The Barnim epidemic strain (t032) and the Rhine-Hesse strain (t002) as well as t768, t6406 and t10973 were found. In two PTS workers, animal-associated MRSA strains (t034) were found even though the participants had reported no animal contact on the questionnaire. Community-associated MRSA was not identified.

## Discussion

Our study on the occupational risk of exposure of PTS and taxi drivers who provide patient transport services is the first to provide data on the prevalence of MRSA in these professions in the greater Hamburg area. The reported results of 3.2% and 0.98% indicate low prevalence rates. No comparative data from Germany are available. 

Two studies on the prevalence of MRSA in PTS were carried out in the US. In their cross-sectional study, Orellana et al. [3] examined PTS in Ohio as well as potential risk factors. The MRSA prevalence was 4.6% (13/280). The risk factors they found were insufficient hand disinfection after glove use (OR 10.51; 95% CI 2.54–43.45) and low frequency of hand washing (<8 times per shift) (OR 4.20; 95% CI 1.02–17.27). In another study, Miramonti et al. [6] investigated and compared PTS with more than 6 months of experience and trainees with less than 2 months of training. Contrary to expectations, no significant differences in MRSA prevalence were found. The rate was 5.3% for the trainees and 4.5% for the PTS.

Authors from Germany evaluated the pathogen load in vehicles and on medical devices [4], [5]. They found that the pathogen load in the vehicles for non-qualified patient transport was higher than in the qualified patient transport. For example, more *cocci* pathogens were found on straps, headrests and door handles. No MRSA was found in the vehicles that provided qualified patient transport. However, in every fourth vehicle of the non-qualified patient transport, MRSA was detected on the straps, door handles, and headrests. However, this is limited by the fact that only non-qualified transport services are used for MRSA patients in Frankfurt, where the study was conducted. Guidelines for good hygiene to prevent the spread of infection are included in the legislation for rescue services in the individual German states. However, no such guidelines exist for non-qualified patient transport services [4].

In the SEKURE study carried out in the Ulm area, the pathogen load of ambulances and patient transport services was also investigated. Tests using contact slides were carried out on medical devices such as ECGs, oxygen saturation clips, and blood pressure cuffs as well as on patient stretchers, handles, etc. MRSA was present more often in emergency service ambulances 1.1% (n=16/1.502) than in patient transport vehicles 0.3% (n=2/634) [5].

Eibicht and Vogel investigated the pathogen load in the vehicles after the transport of MRSA patients. Contamination was found on surfaces that were in direct contact with the patient, such as the headrest and handles of the stretcher. Other surfaces were not affected. Transport time had no effect on contamination [7].

Due to the pathogen load in the vehicles, no immediate conclusions can be drawn about the risk of infection for employees and patients. However, it does show the necessity of cleaning/disinfection measures for surfaces. This applies to patient transport as a whole [4], [8] because transmission can also occur via contaminated surfaces as a study from England shows [9]. In our study, 97% of the employees of the qualified patient transport said that they disinfect their hands after patient contact. Work clothes as potential sources of spreading pathogens are often changed at the end of a work shift or in the event of contamination. Questions about the use of the vehicles and the handling of medical devices were not asked.

The KRINKO recommendations for emergency medical services and patient transport deal with basic hygiene measures and stress the importance of strict adherence while transporting MRSA patients in order to prevent transmission to personnel. This includes personnel disinfecting their hands, the cleaning and disinfection of contact surfaces, proper preparation of medical devices and proper waste disposal [8]. In addition, it recommends further measures, such as sharing information on infection status, wearing a face mask when working with the patient, wearing a protective gown, etc.

MRSA prevalence among qualified patient transport services is somewhat higher than that of the outpatient and inpatient elderly care sector [10]. This may be due to missing information about MDRO colonization in patients who are to be transported. Employees are often not aware of the infection status of the patient and as a result, adequate safety precautions cannot be taken. In terms of infection prevention, however, sharing information about multidrug-resistant organisms is important for everyone concerned in order to ensure optimal patient care and employee protection. In 2005, KRINKO recommended that appropriate instructions should be communicated with regard to the transfer and transport of patients so that adequate protective and hygiene measures can be taken [11]. In order to improve the sharing of information, MDRO information sheets are now being used in some German states. This step, together with the hygiene measures, can help to reduce the risk of infection during patient transport. 

There are no hygiene regulations that govern non-qualified patient transport. However, increasing awareness of the potential infection risks and associated protective measures among drivers should be considered as well as having hand disinfectant in the car if necessary.

Clear regulations regarding MDROs are important for employees who deal with patients or infectious material. For the prevention of transmission of pathogens from patient to employee, such regulations are defined by section 5 of the German Occupational Health and Safety Act, which contains the employers’ duties to provide a safe and healthy workplace. Further specifications regarding these risk assessments are listed in the German Technical Rules for Biological Agents (TRBA). For the healthcare sector, the TRBA 250 contains protective measures with regard to the risk of infection, such as hygienic hand disinfection, protective clothing, and surface disinfection [12].

### Limitations

There was an ongoing problem in this study regarding willingness to participate. This was especially evident when recruiting organisations and/or facilities. Despite written and telephone contact, flyer distribution and sharing information via the MRE-Netzwerk Hamburg, motivating the personnel in charge to participate was difficult. However, we can only speculate about the reasons. The reluctance of employers to agree to MRSA screening is mainly due to the fear of numerous positive results. The worry that MRSA-positive employees would increasingly take sick leave underscores the concern over the pre-existing shortage of personnel in this sector. Uncertainty regarding the proper handling of MRSA-colonized employees and the consequences in terms of the vehicles used for patient transport, for example, may also be contributing factors. In addition, the fear of greater organizational effort required might also be partly responsible for the refusal to participate. It is therefore likely that the results were distorted due to a selection bias. Coupled with low participation rates, an underestimation of the actual MRSA risk cannot be ruled out.

## Conclusions

Until now, little was known about the risk of occupational exposure to MRSA colonization among PTS personnel. This study made it possible to successfully determine the rate of MRSA among EMS personnel and taxi drivers providing patient transport services in Hamburg and to obtain a good picture of the situation concerning occupational MRSA contamination. The prevalence data are low in all areas and indicate a rather low risk of infection. Statements on the success of decolonization are unreliable due to the small number of cases. A good infection control at the facilities is highly recommendable and the employees should acquire in-depth knowledge of infection prevention to improve the compliance with basic hygiene measures such as hand disinfection and personal protective measures. In patient transport, information and communication about the infection status of the patient are important in order to take suitable protective measures. There is room for improvement in this area. The use of a transfer form containing all relevant information is an important step for the prevention of occupational infections during patient transport.

## Notes

### Competing interests

The authors declare that they have no competing interests.

## Figures and Tables

**Table 1:**
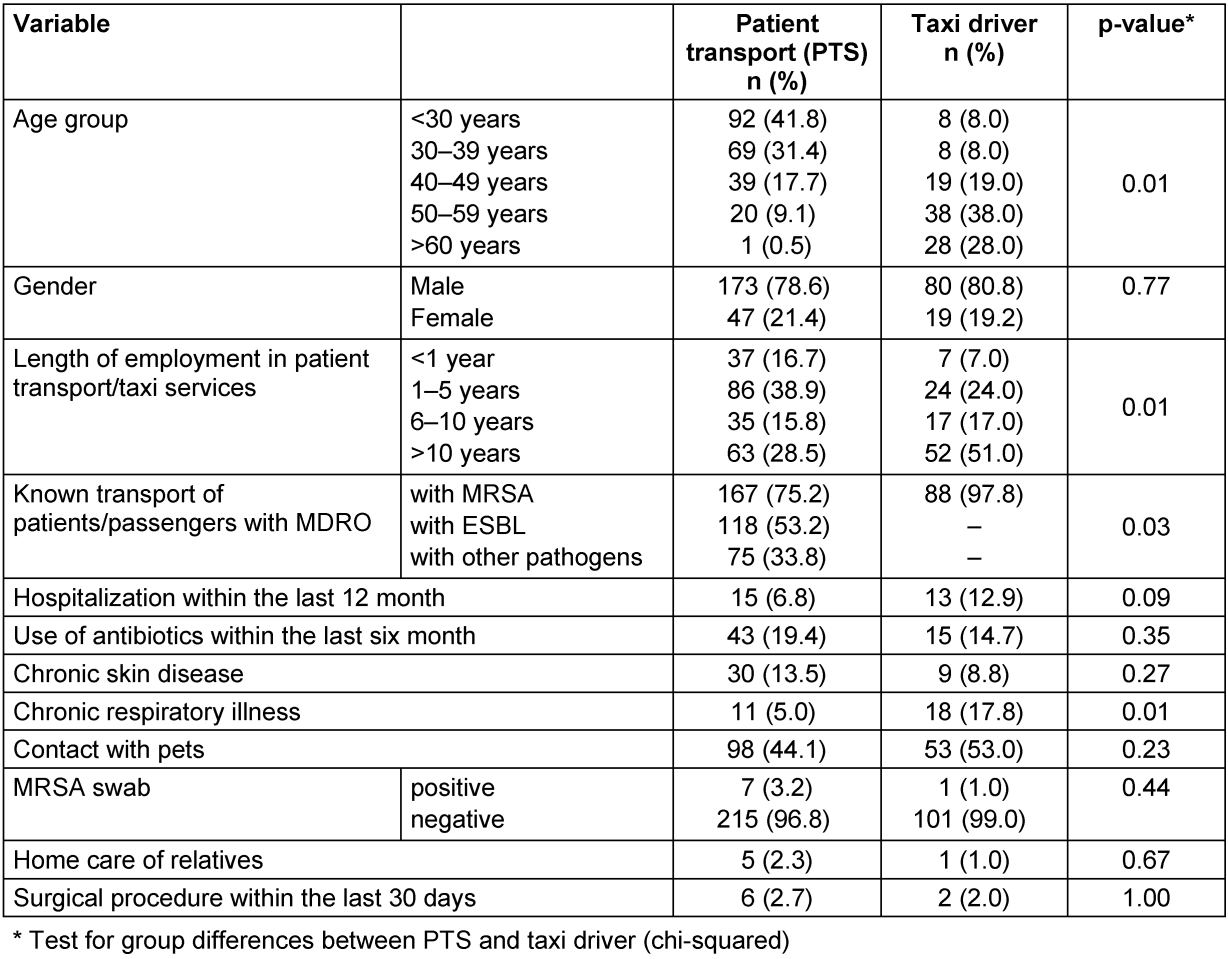
Description of the study population (patient transport n=222, taxi n=102)

**Table 2:**
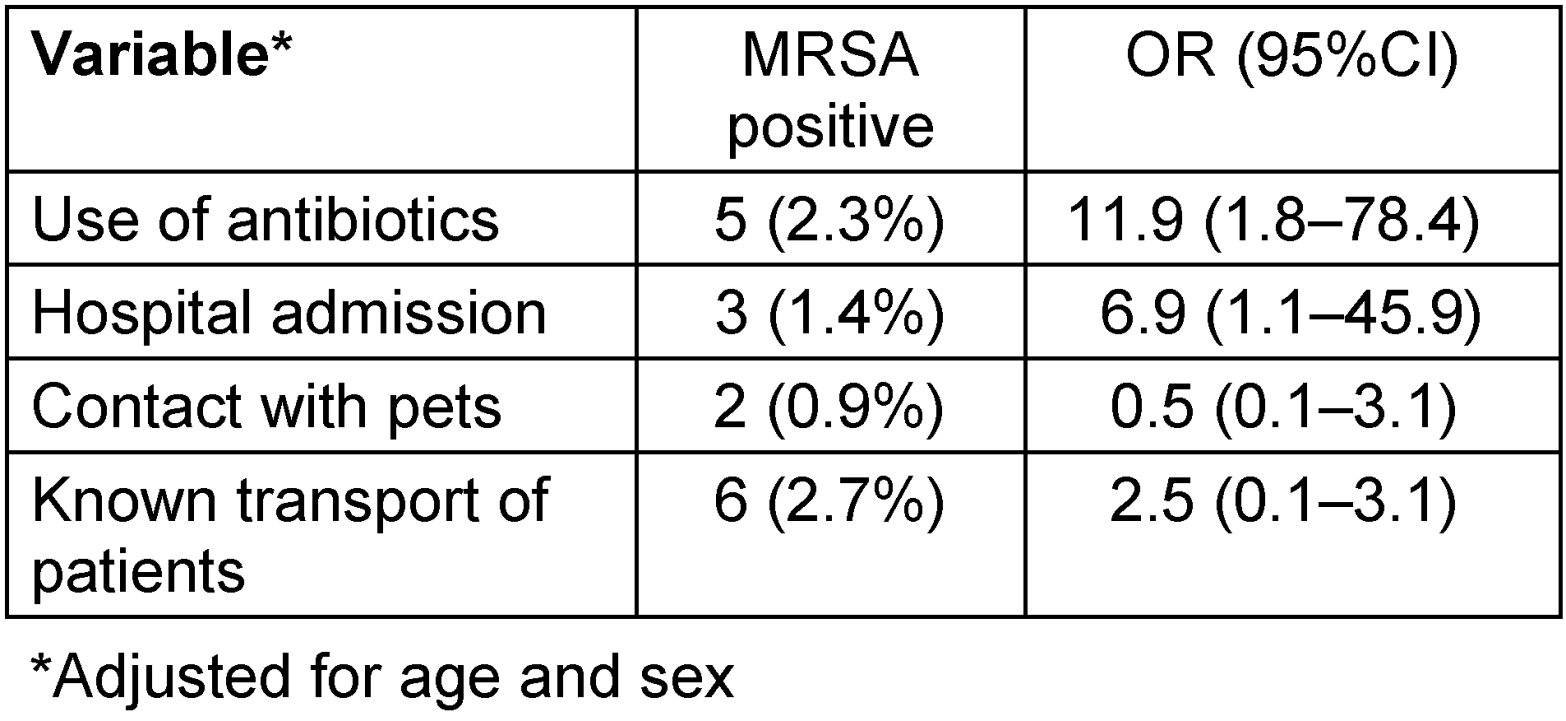
Frequencies and adjusted odds ratios (OR) including 95% confidence intervals (95% CI) for covariates associated with positive MRSA test

## References

[1] Ross B, Spors J, Oberndörfer D, Engelberg H, Popp W (2013). Hygiene in Krankentransport und Rettungsdienst – Empfehlungen unter besonderer Berücksichtigung des Personalschutzes. Hyg Med.

[2] Dulon M, Lisiak B, Wendeler D, Nienhaus A (2015). Berufsbedingte Infektionskrankheiten bei Beschäftigten im Gesundheitsdienst 2014. Zbl Arbeitsmed.

[3] Orellana RC, Hoet AE, Bell C, Kelley C, Lu B, Anderson SE, Stevenson KB (2016). Methicillin-resistant Staphylococcus aureus in Ohio EMS Providers: A Statewide Cross-sectional Study. Prehosp Emerg Care.

[4] Erk G, Brandt C, Heudorf U (2013). Mikrobielle Belastung und multiresistente Erreger im qualifizierten und nichtqualifizierten Krankentransport. Hyg Med.

[5] Wildermuth S, Stahl W, Dirks B, Hafner S, Wepler M, von Baum H (2013). Die Ulmer SEKURE-Studie: Untersuchung der Erregerbelastung im Rettungsdienst – Eine Bestandsaufnahme. Hyg Med.

[6] Miramonti C, Rinkle JA, Iden S, Lincoln J, Huffman G, Riddell E, Kozak MA (2013). The prevalence of methicillin-resistant staphylococcus aureus among out-of-hospital care providers and emergency medical technician students. Prehosp Emerg Care.

[7] Eibicht SJ, Vogel U (2011). Meticillin-resistant Staphylococcus aureus (MRSA) contamination of ambulance cars after short term transport of MRSA-colonised patients is restricted to the stretcher. J Hosp Infect.

[8] (2014). Empfehlung der Kommission für Krankenhaushygiene und Infektionsprävention (KRINKO) beim Robert Koch-Institut. Empfehlungen zur Prävention und Kontrolle von Methicillin-resistenten Staphylococcus aureus-Stämmen (MRSA) in medizinischen und pflegerischen Einrichtungen. Empfehlungen zur Prävention und Kontrolle von Methicillin-resistenten Staphylococcus aureus-Stämmen (MRSA) in medizinischen und pflegerischen Einrichtungen. Bundesgesundheitsblatt Gesundheitsforschung Gesundheitsschutz.

[9] Price JR, Cole K, Bexley A, Kostiou V, Eyre DW, Golubchik T, Wilson DJ, Crook DW, Walker AS, Peto TEA, Llewelyn MJ, Paul J, Modernising Medical Microbiology informatics group (2017). Transmission of Staphylococcus aureus between health-care workers, the environment, and patients in an intensive care unit: a longitudinal cohort study based on whole-genome sequencing. Lancet Infect Dis.

[10] Peters C, Dulon M, Kleinmüller O, Nienhaus A, Schablon A (2017). MRSA Prevalence and Risk Factors among Health Personnel and Residents in Nursing Homes in Hamburg, Germany - A Cross-Sectional Study. PLoS ONE.

[11] (2005). Infektionsprävention in Heimen. Empfehlung der Kommission für Krankenhaushygiene und Infektionsprävention beim Robert Koch-Institut (RKI). Bundesgesundheitsblatt Gesundheitsforschung Gesundheitsschutz.

[12] Bundesanstalt für Arbeitsschutz und Arbeitsmedizin / Ausschuss für biologische Arbeitsstoffe (2014). TRBA 250 – Biologische Arbeitsstoffe im Gesundheitswesen und in der Wohlfahrtspflege. http://www.baua.de/de/Themen-von-A-Z/Biologische-Arbeitsstoffe/TRBA/pdf/TRBA-250.pdf?__blob=publicationFile.

